# Gout and an Animal Diet

**Published:** 1882-03

**Authors:** 


### GOUT AND AN ANIMAL DIET.

The theory, which is widely accepted, that high living, and especially rich animal food, is, with insufficient exercise, the great factor in causing gout, has to meet a good many objections. It has lately been found that gout appears in some of the lower animals when fed upon a purely vegetarian diet. This is notably the case with parrots, according to observations of M. Meguin. In a number of these animals he has found well developed gouty joints.

